# A Viral mRNA Motif at the 3′-Untranslated Region that Confers Translatability in a Cell-Specific Manner. Implications for Virus Evolution

**DOI:** 10.1038/srep19217

**Published:** 2016-01-12

**Authors:** Manuel Garcia-Moreno, Miguel Angel Sanz, Luis Carrasco

**Affiliations:** 1 Centro de Biología Molecular “Severo Ochoa” (CSIC-UAM), Madrid, Spain

## Abstract

Sindbis virus (SINV) mRNAs contain several motifs that participate in the regulation of their translation. We have discovered a motif at the 3′ untranslated region (UTR) of viral mRNAs, constituted by three repeated sequences, which is involved in the translation of both SINV genomic and subgenomic mRNAs in insect, but not in mammalian cells. These data illustrate for the first time that an element present at the 3′-UTR confers translatability to mRNAs from an animal virus in a cell-specific manner. Sequences located at the beginning of the 5′-UTR may also regulate SINV subgenomic mRNA translation in both cell lines in a context of infection. Moreover, a replicon derived from Sleeping disease virus, an alphavirus that have no known arthropod vector for transmission, is much more efficient in insect cells when the repeated sequences from SINV are inserted at its 3′-UTR, due to the enhanced translatability of its mRNAs. Thus, these findings provide a clue to understand, at the molecular level, the evolution of alphaviruses and their host range.

Viral RNA genomes contain several elements that participate in the regulation of a number of viral functions. In positive-stranded RNA viruses, sequences such as Internal Ribosome Entry Site (IRES) elements, located at the 5′ untranslated region (UTR) of viral mRNAs, are involved in mRNA translation^1^,^2^. IRES elements have been described in animal and plant viruses, and also in cellular mRNAs^3^,^4^. In some plant viruses with RNA genomes, several sequences confer cap-independent translation^5^. These sequences are known as CITE (Cap-Independent Translation Element) and can be found in different regions of the genome, although they are most frequently located at the 3′-UTR. It is thought that the main function of the CITE is to recruit components implicated in translation, such as the eIF4F complex, which is thereafter transferred to the 5′ leader sequence^6^,^7^. To our knowledge, CITEs have not yet been described in RNA genomes from animal viruses. In addition to these elements, long-range interactions between the 5′ and 3′ regions of RNA from positive-stranded viruses have been shown to be involved in RNA replication, transcription or translation^8^.

Efficient translation of eukaryotic mRNAs requires their interaction with a number of initiation factors (eIFs) that can participate in mRNA circularization^9^,^10^,^11^. Thus, eIF4E, a component of the eIF4F complex, binds to the cap structure present at the 5′ end of mRNAs, whereas poly(A) binding protein (PABP) interacts with the poly(A) tail that is located at the 3′ end. Joining of eIF4G, another component of the eIF4F complex, to PABP may confer mRNA circularization^12^,^13^. The possibility that this circularization facilitates recycling of translation components has been raised, although this idea has been challenged^14^.

Sindbis virus (SINV) is a prototypic member of the *Togaviridae* family. The 11.7-kb genome of SINV contains an RNA molecule of positive polarity^15^. During virus replication, the negative RNA strand is synthesized, constituting the template to generate the genomic and subgenomic viral mRNAs (gmRNA and sgmRNA, respectively). Thus, two viral mRNAs are translated in SINV-infected cells, both containing a cap-structure at their 5′ end and a poly(A) tail at the 3′ end. The gmRNA is translated early following virus entry and delivery of the genome into the cytoplasm and gives rise to the non-structural proteins (nsPs), which are involved in RNA replication and transcription^16^. The sgmRNA directs the synthesis of viral structural proteins later in infection, when cellular translation has been drastically arrested. Notably, the mechanism of sgmRNA translation involves a scanning of the leader sequence by the preinitiation complex, without the participation of several eIFs^17^.

A number of elements have been distinguished in sgmRNA that make it particularly efficient for translation during infection. Among these is the hairpin-loop structure, also known as DLP, located 24 nucleotides downstream of the AUG initiation codon that is required to translate the sgmRNA in the absence of active eIF2 in SINV-infected mammalian cells^18^,^19^,^20^. However, phosphorylation of eIF2α is not observed in SINV-infected insect cells, which do not encode the Protein Kinase R (PKR), and in infected mammalian PKR knock-out cells^17^,^18^,^21^. In addition, the leader sequence of sgmRNA has been shown to be involved in the translatability of sgmRNA and in the shut-off of host translation^22^.

The SINV 3′-UTR is rather long (323 nt) and contains a conserved 19 nt sequence that, together with at least 11 nt of the poly(A) tail, form part of the promoter to synthesize minus-stranded RNA^23^,^24^. A U-rich sequence of about 60 nt is found before this conserved region, which interacts with the host protein HuR and is involved in mRNA stabilization during alphavirus infection^25^,^26^,^27^. Additionally, there are three repeated stem-loop structures that are present not only in alphaviruses, but also in other arthropod borne viruses (arboviruses)^24^,^28^,^29^. These elements, as well as the U-rich domain, may also contribute to the repression of deadenylation of viral mRNAs^30^. Deletion of most of the 3′-UTR, whilst retaining the 19 nt conserved sequence, decreases the efficiency of SINV replication in mosquito cells relative to chicken cells^31^. Mutagenesis of these regions has different effects on viral replication in mice and in cultured murine cells^32^, which are dependent not only on the species, but also on the tissue analyzed; however, their exact functioning remains unclear.

In the present work, we examined the role of these three repeated sequences at the 3′-UTR of SINV mRNAs during the virus life cycle in mammalian and insect cells. Mutation of the three repeated regions had little effect for the translation of both viral mRNAs in mammalian cells; however, protein synthesis directed by these two mRNAs was impeded in mosquito cells. Furthermore, the addition of these SINV repeated sequence elements to the short 3′-UTR of Sleeping disease virus (SDV)^33^,^34^,^35^,^36^, an alphavirus for which an invertebrate vector has not been identified, potently increased its replication in insect cells. To our knowledge, this motif present at the 3′-UTR of SINV mRNAs constitutes the first example of an element that confers translatability to mRNAs from an animal virus in a cell-specific manner.

## Results

### Mutation of the motifs present at the 3′-UTR of SINV mRNAs. Effects on gmRNA translation

**Figure 1:**
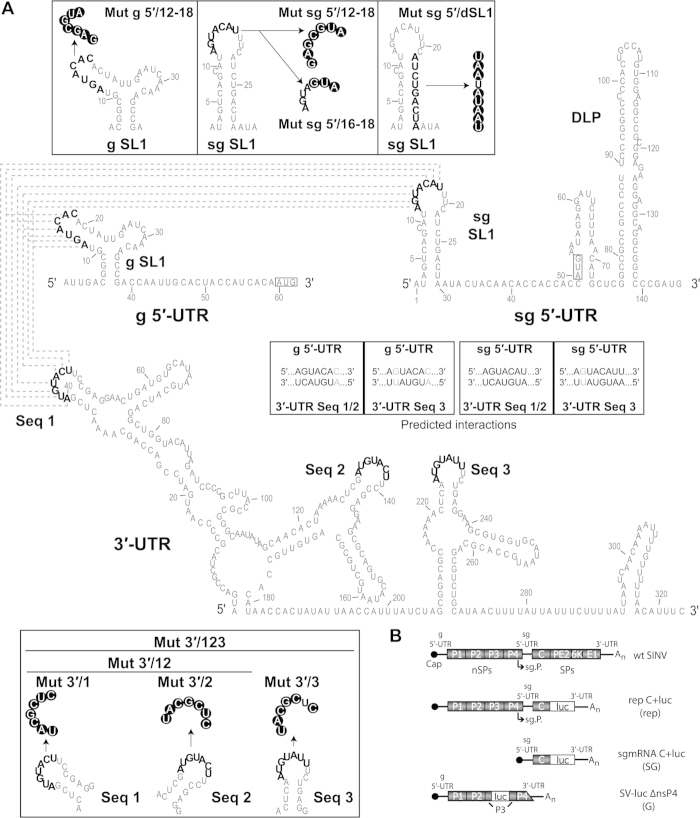
Theoretical model for the interaction between the 5′ and 3′-UTR of SINV g- and sgmRNA. (**A**) Sequence and secondary structure of the 5′ and 3′ ends of SINV g- and sgmRNA predicted by RNAfold. The nucleotides potentially involved in the predicted long-range RNA-RNA interactions between the 5′ and 3′-UTRs (dotted lines) are shown in bold. The nucleotides replaced to obtain the different mutants are indicated in white within black circles. The AUG initiation codons are boxed. (**B**) Schematic diagram of the SINV genome (wt SINV) and the different constructs used in translation assays: the rep C+luc replicon, the subgenomic mRNA C+luc and the non-replicating genomic mRNA SV-Luc ∆nsP4 (abbreviated as “rep”, “SG” and “G”, respectively). Abbreviations: sg, subgenomic; SL, stem-loop; UTR, untranslated region; Mut, mutant; Seq, sequence; g, genomic; nsPs, non-structural proteins; sPs, structural proteins; sg.P., subgenomic promoter.

**Figure 2:**
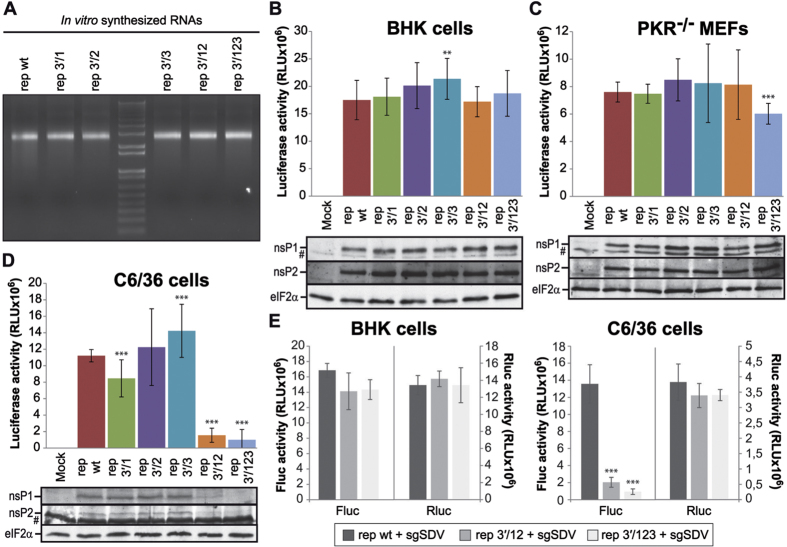
Protein synthesis in cells transfected with SINV replicons harboring mutated 3′-UTRs. (**A–D**) SINV replicons obtained by *in vitro* transcription with T7 RNA polymerase (**A**) were transfected into BHK (**B**), *PKR*^−/−^ MEFs (**C**) and C6/36 (**D**) cells using Lipofectamine 2000. Cells were harvested at 7, 8 and 9 hpt, respectively, to analyze luciferase activity. The data are mean ± SD of three independent experiments performed in triplicate (*upper panels*). In parallel, the accumulation of the non-structural proteins nsP1 and nsP2, as well as total eIF2α as loading control, was evaluated by immunoblotting using specific antibodies (*lower panels*). #indicates nonspecific bands. (**E**) BHK (*left panel*) and C6/36 (*right panel*) cells were co-transfected with *in vitro* transcribed rep wt, rep 3′/12 or rep 3′/123 plus a *Renilla* luciferase reporter mRNA (sgSDV wt, see Fig. 9 and Supplementary Table S1) using Lipofectamine 2000. Cells were processed for the measurement of firefly and *Renilla* luciferase produced from the replicons (at 7 hpt in BHK cells and at 9 hpt in C6/36 cells) and the reporter mRNA (at 4 hpt), respectively. Values of firefly (Fluc, *left Y axis*) and *Renilla* (Rluc, *right Y axis*) luciferase activity are the mean ± SD of triplicate experiments.

We observed that some nucleotides present at the loop of the stem-loop structures (SL1) located at the 5′-UTRs of g- and sgmRNA are complementary to nucleotides situated in three loops (Seq 1, Seq 2 and Seq 3) of the SINV genome 3′-UTR (see scheme in Fig. 1A). Previous observations using electron microscopy showed that the SINV genome circularizes^37^,^38^, and the 5′ and 3′ ends of SINV gmRNA have been proposed to interact, contributing to SINV replication^39^. To evaluate the function of these sequences, we first employed the SINV replicon rep C+luc (abbreviated as “rep”), which contains the entire coding region of capsid (C) protein fused to the complete firefly luciferase gene (Fig. 1B). Translation of the sgmRNA derived from this replicon produces a fusion protein that is cleaved at the C carboxyl-terminus immediately after its generation due to the autocatalytic activity of C, releasing C and luciferase proteins. Five different variants of the replicon were constructed (Fig. 1A), bearing mutations in the sequences 1, 2 and 3 of the 3′-UTR individually (rep 3′/1, rep 3′/2 and rep 3′/3, respectively) or in combination 1+2 (rep 3′/12) or 1+2+3 (rep 3′/123). Confidence in the secondary structure predictions of SINV wt and mutated 3′-UTRs is presented in Supplementary Fig. S1A. Replicons were synthesized *in vitro* by T7 RNA polymerase (Fig. 2A) and were transfected into two mammalian cell lines (BHK cells and *PKR*^−/−^ MEFs). These cell lines differ in the phosphorylation status of eIF2α after SINV infection. Consequently, SINV induces strong phosphorylation of eIF2α in infected BHK cells, while no increase in phosphorylation is observed in *PKR*^−/−^ MEFs^17^. Following transfection, gmRNA translation and RNA replication/transcription occurs, giving rise to sgmRNA, and the activity of luciferase produced from these sgmRNAs can be measured. As observed in Fig. 2B,C (upper panels), none of the mutants gave rise to variations in luciferase activity higher than 20% with respect to control rep wt. The levels of two early viral proteins, nsP1 and nsP2, were also similar in all the replicons tested (Fig. 2B,C, lower panels). Thus, mutation of the 3′-UTR of SINV rep has little effect on the synthesis of proteins directed by g- and sgmRNA in mammalian cells. Because the 3′-UTR of Chikungunya virus has been suggested to be important in selective adaptation to mosquito, but not to mammalian cells^40^,^41^, it was of interest to examine the effect of 3′-UTR mutations in this additional natural alphavirus host. We transfected 3′-UTR variant replicons into *Aedes albopictus* C6/36 cells and measured luciferase activity. Compared with rep wt, luciferase synthesis in C6/36 cells was marginally affected by transfection with rep 3′/1 (24% inhibition), rep 3′/2 (9% stimulation) and rep 3′/3 (26% stimulation) at 8 hours post-transfection (hpt) (Fig. 2D, upper panel). In contrast, the production of luciferase was profoundly reduced when double (rep 3′/12, 86% inhibition) or triple (rep 3′/123, 91% inhibition) mutations were introduced (Fig. 2D, upper panel). As a control for transfection efficiency, BHK and C6/36 cells were co-transfected with rep wt, rep 3′/12 or rep 3′/123 and a reporter mRNA encoding the *Renilla* luciferase. As expected, all the replicons tested in BHK cells produced equivalent amounts of firefly luciferase (Fig. 2E, left panel), whereas luciferase synthesis was strongly reduced when rep 3′/12 and rep 3′/123 were transfected in insect cells (85 and 93% inhibition, respectively, as compared to control rep wt, Fig. 2E, right panel). In both cell lines, values of *Renilla* luciferase activity from the reporter mRNA were similar after co-transfection with the different replicons, suggesting equal transfection efficiencies.

**Figure 3:**
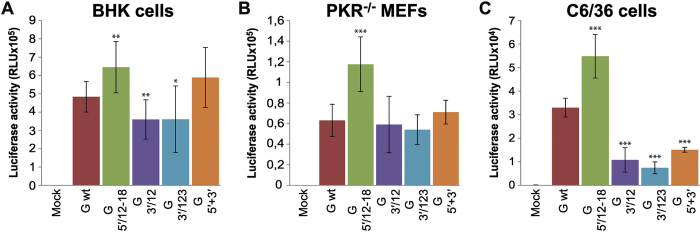
Influence of UTRs in the translational efficiency of SINV gmRNA in different cell types. (**A–C**) BHK (**A**), *PKR*^−/−^ MEFs (**B**) and C6/36 (**C**) cells were transfected with *in vitro* transcribed (SP6 RNA polymerase) non-replicating SINV gmRNAs (“G”) using Lipofectamine 2000. At 4 hpt, cells were collected and lysed for luciferase measurement. Values of luciferase activity represent the mean ± SD of three experiments carried out in triplicate.

Different steps of the replication cycle may be involved in this robust reduction of luciferase activity, including gmRNA translation, SINV RNA replication/transcription or sgmRNA translation. Since the translation of gmRNA is the first event following the delivery of the SINV genome into the cell, we analyzed the production of nsPs. In good agreement with the luciferase measurements, synthesis of nsP1 and nsP2 was strongly blocked in rep 3′/12 and rep 3′/123 transfected C6/36 cells, and bands corresponding to these proteins were barely detected by Western blotting (Fig. 2D, lower panel). In contrast, nsPs were produced at control levels in rep 3′/1, rep 3′/2 and rep 3′/3 transfected cells (Fig. 2D, lower panel). To further assess whether the 3′-UTR may have a role in regulation of translation of SINV gmRNA in insect cells, we used the construct SV-Luc ∆nsP4 (abbreviated as “G”), which contains the firefly luciferase gene embedded in the nsP3 sequence and is unable to replicate due to the deletion of most of the coding sequence for nsP4, the viral RNA-dependent RNA polymerase (Fig. 1B). This construct allows the direct analysis of translation of the input gmRNA. Several variants of this gmRNA were designed: G 3′/12 and G 3′/123 with mutated 3′-UTR, G 5′/12–18 with altered SL1 in 5′-UTR, and G 5′+3′ with compensatory base changes in both 5′ and 3′-UTRs (with all the three mutated segments at the 3′ end), restoring the potential long-range interaction (Fig. 1A). Transfection of BHK cells with G 5′/12–18 resulted in an increase in luciferase synthesis compared with control G wt (133% stimulation, Fig. 3A). This result is consistent with previous data that revealed an increase in translational efficiency upon mutation of g SL1^42^. Mutants of the 3′-UTR exhibited a small decrease in luciferase activity (25% in G 3′/12 and G 3′/123), and the mutant G 5′+3′ did not change significantly in comparison to G wt. Luciferase activity values from G 5′/12–18 were 186% compared with G wt in *PKR*^−/−^ MEFs, whereas G 3′/12, G 3′/123 and G 5′+3′ were as efficient as the control (Fig. 3B). Translation of G 5′/12–18 was also increased relative to wt in C6/36 cells (166%, Fig. 3C). However, mutations introduced into the 3′-UTR had a clear inhibitory effect on translation in C6/36 cells, in good agreement with the previous results (see Fig. 2D). Accordingly, when G 3′/12, G 3′/123 and G 5′+3′ were transfected, luciferase activity was reduced by 67%, 77% and 54%, respectively, relative to G wt (Fig. 3C).

Collectively, these results revealed that the repeated sequences present at the 3′-UTR of SINV genome may regulate the translation of gmRNA selectively in mosquito, but not in mammalian cells. Moreover, the predicted kissing-loop interaction between the 5′ and 3′-UTRs of gmRNA might not be crucial since mutation of the nucleotides located at the genomic 5′-UTR had no negative effects in any of the cell lines analyzed. Alternatively, it is possible that, in addition to the potential kissing-loop interaction, other events may be necessary to increase the translation of SINV gmRNA.

### Importance of the 5′ leader sequence of SINV sgmRNA for translation in replicating cells

**Figure 4:**
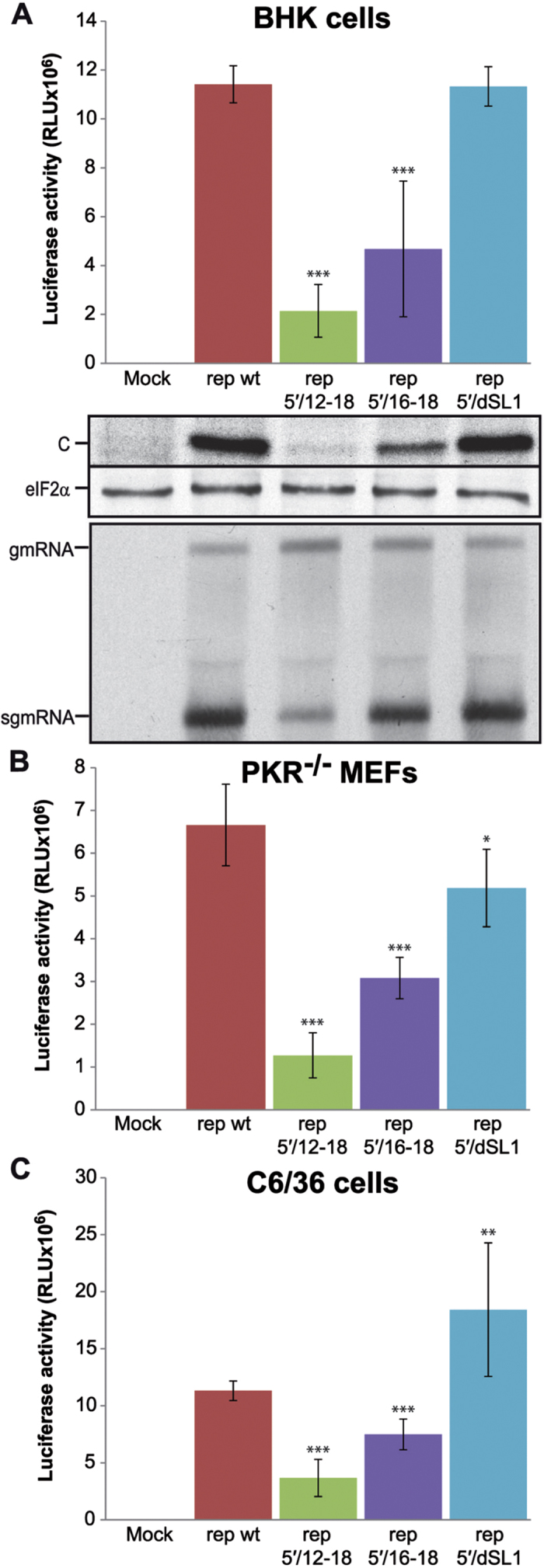
Translation and RNA synthesis in different cells after transfection with replicons bearing altered sgmRNA 5′-UTR. (**A**) BHK cells were transfected with *in vitro* transcribed SINV replicons using Lipofectamine 2000. Cells were recovered in luciferase lysis buffer at 7 hpt and luciferase activity was measured (*upper panel*). Synthesis of protein C was analyzed by radioactive labelling with [^35^S]Met/Cys from 6 to 7 hpt, followed by separation on SDS-PAGE and autoradiography; in parallel, total eIF2α was detected by western blotting as loading control (*middle panel*). Viral RNAs were detected by [^3^H]uridine incorporation from 3 to 7 hpt and separation in agarose gels, as described in *Methods* (*lower panel*). (**B,C**) *PKR*^−/−^ MEFs (**B**) and C6/36 cells (**C**) were transfected as in (**A**). Luciferase activity was determined at 6 and 8 hpt, respectively. Values of luciferase activity represent the mean ± SD of three experiments done in triplicate.

As indicated above, the complementary sequences of seven nucleotides in the 5′ and 3′-UTRs of SINV sgmRNA (Fig. 1A) might play a role during infection. Indeed, mutation of the 3′-UTR of rep had little consequence for translation in BHK and *PKR*^−/−^ cells, but this region was important for gmRNA translation in insect cells. In addition, it is still possible that these sequences are also relevant for sgmRNA functioning. Therefore, we evaluated the importance of the hairpin structure present at the 5′-UTR of sgmRNA using SINV replicons with mutated sg SL1 (Fig. 1A and Supplementary Fig. S1B). In the construct rep 5′/12–18, we substituted the loop-forming nucleotides 12 to 18 by site-directed mutagenesis. Nucleotides 1–5 of the sgmRNA 5′-UTR are necessary for the activity of the internal promoter^43^, but the number of nucleotides required for the full activity of this promoter remains unknown, although the first 14 nucleotides have been proposed to be important^44^,^45^. For this reason, we also mutated only nucleotides 16 to 18 (rep 5′/16–18). The variant rep 5′/dSL1 contains mutations in nucleotides 22 to 30 that disrupts the hairpin sg SL1, which is conserved among different alphavirus species^46^. These replicons were first assayed in BHK cells. Protein synthesis was strongly inhibited after transfection of rep 5′/12–18, as observed by a robust decrease in luciferase activity (81% reduction compared with rep wt) and a reduction in metabolic labelling of SINV C protein (Fig. 4A, upper and middle panels, respectively). In the case of rep 5′/16–18, luciferase values decreased by 59%, and C protein synthesis was also reduced. Conversely, rep 5′/dSL1 was as efficient as control rep wt in both luciferase activity and C production. However, as mentioned previously, these differences in protein synthesis may be due to the fact that the internal promoter is affected by the mutations introduced into the leader sequence of sgmRNA. Hence, in a parallel experiment, this possibility was tested by analysing viral RNA synthesis. To do this, BHK cells were transfected with the different replicons, treated with actinomycin D at 2 hpt and labeled with [^3^H]uridine from 3 to 7 hpt. Total RNA was then extracted and separated on agarose gels to measure labeled viral RNAs. Interestingly, sgmRNA synthesis was profoundly diminished in replicon rep 5′/12–18, whereas the production of sgmRNA from rep 5′/16–18 and rep 5′/dSL1 was virtually unchanged relative to rep wt (Fig. 4A, lower panel). Therefore, nucleotides 16 to 18 may contribute to the efficient translation of SINV sgmRNA without affecting the efficacy of the internal promoter, whereas the structure of sg SL1 *per se* may not be important for sgmRNA functioning.

Next, luciferase activity was similarly monitored in *PKR*^−/−^ and C6/36 cells transfected with the different replicons as before. Results in *PKR*^−/−^ MEFs were very similar to those obtained in BHK cells: rep 5′/12–18 and rep 5′/16–18 were inhibited by 81% and 54%, respectively, while rep 5′/dSL1 was reduced by only 22% (Fig. 4B). Some differences were observed in mosquito cells. Mutations in the loop of sg SL1 were less detrimental than observed in mammalian cells and luciferase activity from rep 5′/12–18 and rep 5′/16–18 was diminished by 68% and 34%, respectively. Disruption of the stem of sg SL1 (rep 5′/dSL1) led to a 163% stimulation relative to rep wt (Fig. 4C).

Taken together, these findings indicate that nucleotides 12–15 of the sgmRNA 5′-UTR are important for transcription, but nucleotides 16–18, which barely affect RNA synthesis, are also necessary for the efficient translation of this viral mRNA.

### Translation of SINV sgmRNAs and non-viral mRNAs with mutated 5′ and/or 3′-UTRs out of a replication context

**Figure 5:**
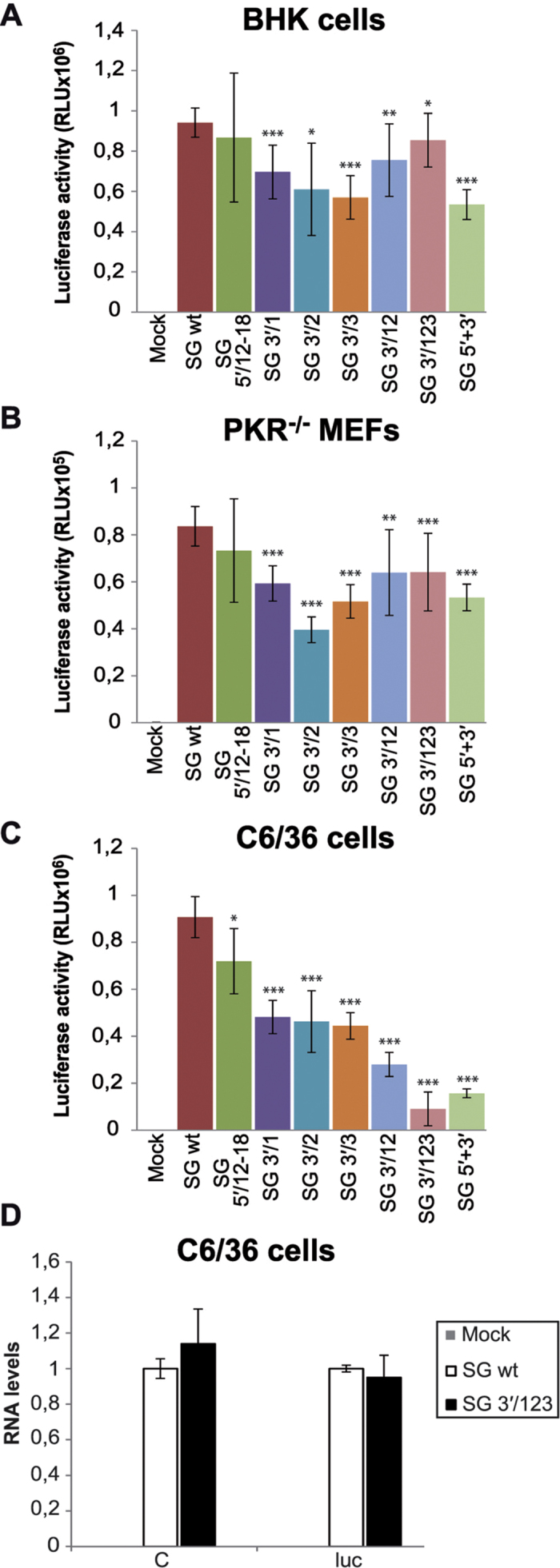
Effect of mutations in the UTRs on translation directed by SINV sgmRNAs transfected into cells. (**A–C**) BHK (**A**), *PKR*^−/−^ MEFs (**B**) and C6/36 (**C**) cells were transfected with *in vitro* transcribed (T7 RNA polymerase) SINV sgmRNAs using Lipofectamine 2000. Cells were recovered in luciferase lysis buffer at 4 hpt and luciferase activity was measured. Values of luciferase activity obtained from the different sgmRNAs are presented as mean ± SD of three independent assays. (**D**) Additionally, total RNA was isolated at 4 hpt from C6/36 transfected cells and used as template to quantify the amount of SG wt and SG 3′/123 by qRT-PCR with C and luc specific primers, as described in *Methods*. The relative RNA levels are represented with respect to SG wt values, and are the mean ± SD of three different experiments.

We next wanted to examine, directly, translation of reporter sgmRNAs without viral RNA replication. To achieve this, several variants of plasmid pT7 C+luc were constructed (see Supplementary Table S1 for description). *In vitro* transcription of template plasmids using T7 RNA polymerase gives rise to sgmRNAs without nsPs and with an identical sequence as those synthesized in cells from the internal promoter (see Fig. 1B, abbreviated as “SG”). As before, different cell lines were transfected with the isolated sgmRNAs and luciferase activity was measured. In contrast to what was observed with replicons rep 5′/12–18 and rep 5′/16–18 (Fig. 4), luciferase values in BHK cells transfected with SG 5′/12–18 were similar to control SG wt (Fig. 5A). Taken together, results from Figs 4 and 5A suggest that nucleotides 12–15 of the subgenomic 5′-UTR are necessary for sgmRNA transcription, whereas nucleotides 16–18 may be important for translation only when the sgmRNA is synthesized by the viral replicase. However, when the isolated sgmRNA is transfected into cells out of a replication context, nucleotides 12–18 of the 5′-UTR have no influence on translation. In this regard, we have reported previously that the translation initiation mechanism of SINV sgmRNA may differ depending on the cellular context^17^,^18^,^19^. Individual mutations of the repeated sequences located at the 3′-UTR resulted in a partial inhibition of luciferase synthesis: 26%, 35% and 39% for SG 3′/1, SG 3′/2 and SG 3′/3, respectively (Fig. 5A). sgmRNAs with 3′-UTRs containing double and triple mutations (SG 3′/12 and SG 3′/123) were only 20% and 9% less efficient than SG wt. Luciferase activity from the additional sgmRNA tested, SG 5′+3′ with compensatory mutations in 5′ and 3′-UTRs, was diminished by 43% compared with the control transfection. Translation in *PKR*^−/−^ MEFs was also studied. Consistent with the results in BHK cells, SG 5′/12–18 was translated to an extent similar to the control in *PKR*^−/−^ MEFs (Fig. 5B). SG 3′/1, SG 3′/2 and SG 3′/3 exhibited a reduction of 29%, 52% and 38% in luciferase production, respectively. There was little inhibition of protein synthesis in SG 3′/12 and SG 3′/123 transfected cells. Furthermore, luciferase synthesis was decreased by 36% in *PKR*^−/−^ MEFs that translated sgmRNA with mutated 5′ and 3′-UTRs (SG 5′+3′, Fig. 5B). In mosquito C6/36 cells, translation of the transfected SG 5′/12–18 was little affected by the modifications in the 5′ leader sequence (21% reduction, Fig. 5C). However, changes in the 3′-UTR had inhibitory effects on protein synthesis. Consequently, luciferase values from SG 3′/1, SG 3′/2 and SG 3′/3 were 46–51% lower than in SG wt. In the case of SG 3′/12, translation was reduced by 69%, while SG 3′/123 was blocked to a similar degree as the corresponding replicon (90%). Protein synthesis directed by SG 5′+3′ was also potently restricted (83%). In parallel, sgmRNA levels were analyzed by quantitative RT-PCR. To do this, SG wt and SG 3′/123 were transfected in C6/36 cells, total RNA was extracted after 4 hours, and the amount of sgmRNA quantified. Results clearly indicated that the amount of wt and 3′/123 sgmRNAs was similar in mosquito cells at 4 hpt (Fig. 5D). Collectively, these findings demonstrate that the repeated regions located at the 3′-UTR of SINV participate directly in sgmRNA translation enhancement selectively in insect cells.

**Figure 6:**
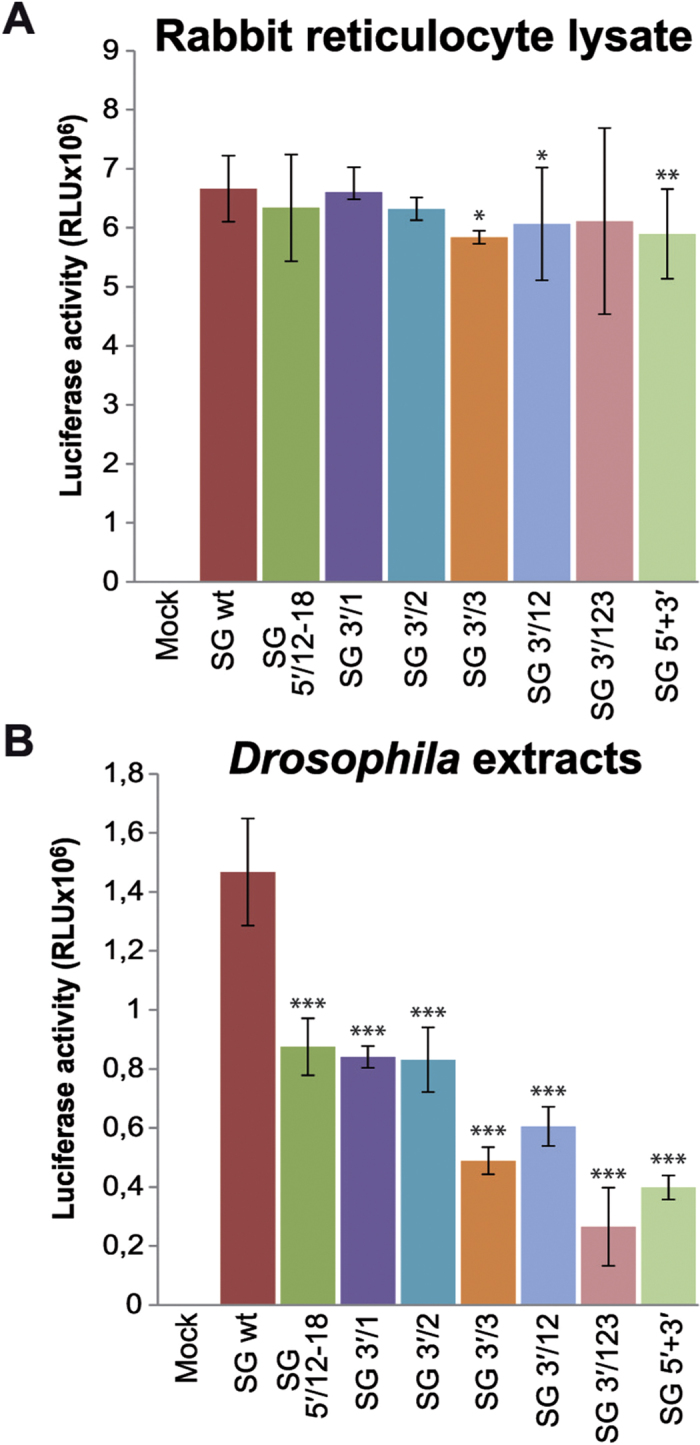
Translation of SINV sgmRNAs in mammalian and insect cell-free systems. (**A,B**) SINV sgmRNAs prepared *in vitro* using T7 RNA polymerase were added to RRL (**A**) or *Drosophila* extracts (**B**) and incubated for 90 min at 30 °C or 25 °C, respectively. Luciferase activity was measured and the results from three independent experiments were plotted as mean ± SD.

The participation of UTRs in SINV sgmRNA translation was also evaluated in cell-free systems. Thus, rabbit reticulocyte lysates (RRL) were programmed with the *in vitro* prepared sgmRNAs described above and luciferase activity was measured. As shown in Fig. 6A, all SG variants yielded similar luciferase levels regardless of whether the 5′-UTR, 3′-UTR, or both, were mutated. *In vitro* translation of these mRNAs was also examined in a cell-free system from *Drosophila melanogaster* extracts^47^. In contrast to the results obtained in RRL, several differences were found in this system. Site-directed mutagenesis of nucleotides 12–18 in sg 5′-UTR resulted in a reduction of sgmRNA translation by approximately 40% (Fig. 6B). Synthesis of luciferase directed by SG 3′/1, SG 3′/2 and SG 3′/3 was decreased by 43%, 43% and 66%, respectively, relative to SG wt. In agreement with the previous findings, SG 3′/12 and SG 3′/123 were inhibited by 59% and 82% with respect to the control. Finally, translation directed by SG 5′+3′ was also potently reduced by 73% (Fig. 6B). Taken together, the results obtained in non-replication contexts support the concept that SINV sgmRNA requires an intact 3′-UTR for efficient translation in insect systems.

**Figure 7:**
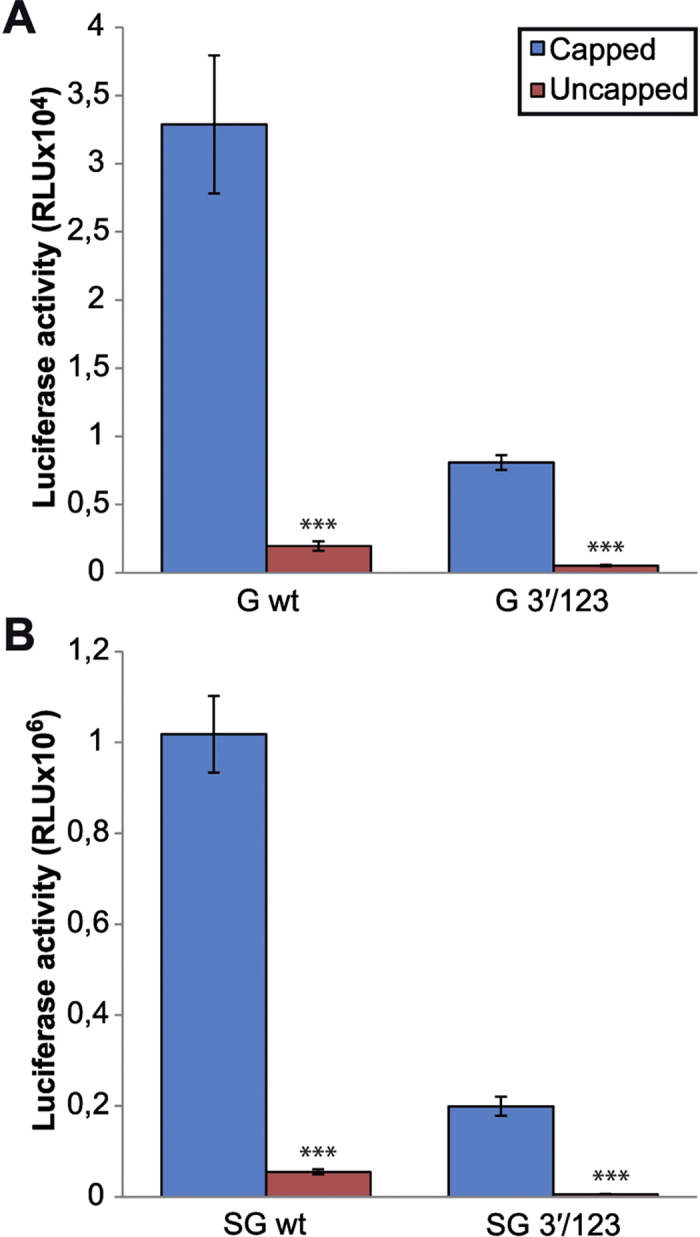
Translation of capped and uncapped SINV g- and sgmRNAs in insect cells. (**A,B**) G and SG in wild type or mutant 3′/123 versions were synthesized by *in vitro* transcription in reactions containing or not the m^7^G(5′)ppp(5′)G cap analog. Next, the corresponding gmRNAs (**A**) or sgmRNAs (**B**) were transfected into mosquito C6/36 cells using Lipofectamine 2000. At 4 hpt, cells were harvested and lysed to measure luciferase activity. The results are represented as the mean ± SD of two independent experiments performed in duplicate.

Because substantial structural similarity exists between the SINV 3′-UTR motif and some elements present in plant viruses that have been described as CITEs^5^, we wished to examine the extent to which the SINV motif behaved in a way similar to that described for an mRNA bearing a CITE. Thus, translation directed by capped or uncapped viral mRNAs (g- and sgmRNA), with or without mutations at the 3′-UTR, was examined in mosquito cells. Translation directed by uncapped g- and sgmRNA was strongly diminished in C6/36 cells, and this inhibition was evidently greater when the three sequences at the 3′-UTR were mutated (Fig. 7). Therefore, the motif present at the 3′-UTR of SINV mRNAs does not mimic the CITEs that confer cap-independence for translation in plant viruses.

**Figure 8:**
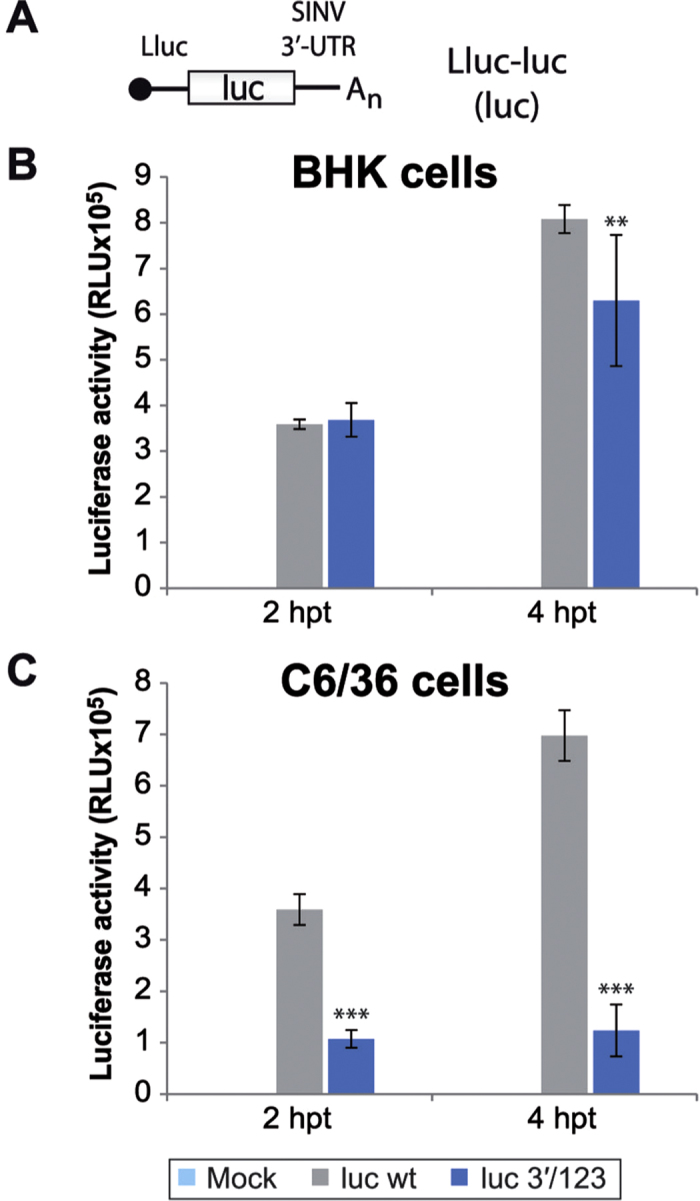
Luciferase synthesis from recombinant mRNAs containing the luc leader sequence and different SINV 3′-UTRs. (**A**) Schematic representation of the mRNAs employed in this experiment. Abbreviations: luc, firefly luciferase; Lluc, luc leader sequence; UTR, untranslated region. (**B,C**) luc wt and mutant luc 3′/123 mRNAs synthesized *in vitro* by T7 RNA polymerase were transfected into BHK (**B**) and C6/36 (**C**) cells using Lipofectamine 2000. At 2 and 4 hpt, cells were lysed and luciferase activity determined. The data are the mean ± SD of triplicate experiments.

To further assess the potential translation enhancing activity of this repeated stem-loop motif, the entire SINV 3′-UTR was located at the 3′-end of a non-viral mRNA. We used a firefly luciferase mRNA bearing the luciferase leader sequence at the 5′-end and the SINV 3′-UTR wt or with mutated sequences in the three loops, designated as luc wt and luc 3′/123, respectively (see scheme in Fig. 8A and specific mutations in Fig. 1A). Transfection of the different *in vitro* synthesized mRNAs into BHK cells gave rise to almost similar values of luciferase activity (only 22% difference at 4 hpt, Fig. 8B). In contrast, luciferase synthesis from luc 3′/123 was considerably lower than from luc wt in mosquito cells (70% and 82% reduction in translation at 2 and 4 hpt, respectively, Fig. 8C). In conclusion, the wt 3′-UTR of SINV confers translatability to a non-viral mRNA in C6/36, but not in BHK cells.

### Evolutionary implications of SINV 3′-UTR repeated sequence elements

**Figure 9:**
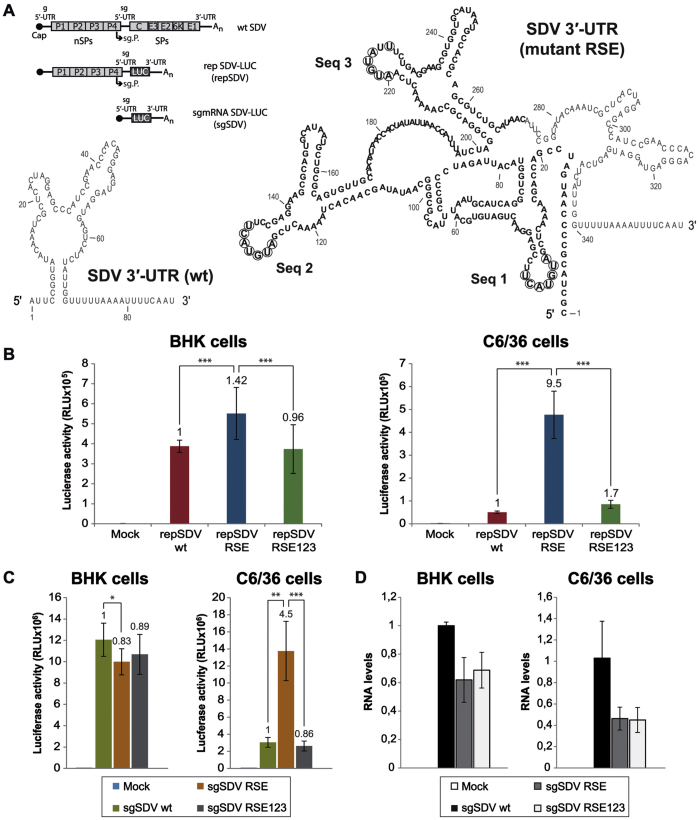
Translation of SDV replicons and *in vitro* synthesized sgmRNAs containing 3′-UTRs with SINV repeated sequences. (**A**) Schematic diagrams of the SDV replicons and sgmRNAs, designated as “repSDV” and “sgSDV”, respectively. See abbreviations in Fig. 1; LUC, *Renilla* luciferase. The RNA secondary structure of the different 3′-UTRs was predicted using the RNAfold WebServer. The region containing SINV repeated sequence elements (RSE), inserted at the beginning of SDV 3′-UTR, is shown in bold. The nucleotides modified to obtain the variant RSE123 (see mutations 3′/123 in Fig. 1A) are circled. (**B,C**) The different SDV replicons (**B**) or the corresponding sgmRNAs synthesized *in vitro* (**C**) were transfected into BHK (*left panels*) and C6/36 (*right panels*) cells using Lipofectamine 2000. At 24 hpt (replicons), or at 4 hpt (*in vitro* made sgmRNAs), cells were processed for the measurement of *Renilla* luciferase activity. (**D**) BHK and C6/36 cells were transfected as in (**C**). At 4 hpt, the amount of sgSDV wt, RSE and RSE123 was quantified by qRT-PCR with *Renilla* luc specific primers. RNA levels are represented relative to sgSDV wt values. The values shown in (**B–D)** are the mean ± SD of three independent experiments carried out in triplicate. The x-fold change of luciferase activity obtained from mutant RNAs relative to wt are indicated in the graphs.

Most alphavirus species belong to the arbovirus group since they can infect both an arthropod and a vertebrate host. Only a few alphaviruses have one vertebrate host and, in principle, do not replicate in insect cells. Among them is SDV, which can infect several salmonid species and is not yet known to have an arthropod vector^34^. Interestingly, SDV contains a short 3′-UTR lacking repeated sequence elements^36^. To test whether the repeated regions present at the 3′-UTR of SINV provide a higher replicating activity to SDV in mosquito cells, we employed a SDV-derived replicon bearing the *Renilla* luciferase gene in place of the viral structural proteins^35^, abbreviated here as repSDV wt (see scheme in Fig. 9A and description in Supplementary Table S1). Two additional constructs were engineered though insertion of SINV elements at the beginning of SDV 3′-UTR: one of them harboring the wt repeated sequences of SINV 3′-UTR (repSDV RSE), and the other containing the same region of SINV but with Seq 1, 2 and 3 mutated simultaneously (repSDV RSE123). The RNA secondary structure and nucleotide sequence of the resulting 3′-UTRs is shown in Fig. 9A. The three RNA replicons were synthesized *in vitro* and transfected in BHK and C6/36 cells, followed by luciferase activity estimation at 24 hpt. Strikingly, in insect cells, the presence of SINV wt repeated sequences in repSDV RSE led to a 9.5-fold increase in luciferase production as compared to repSDV wt, whereas this increase was more modest (1.4-fold) in BHK cells (Fig. 9B). Similar results were observed when luciferase synthesis was measured in both cell lines at increasing times post-transfection (Supplementary Fig. S2A). Moreover, mutation of Seq 1 + Seq 2 + Seq 3 caused a dramatic decrease (82%) in luciferase synthesis in mosquito C6/36 cells, and a much smaller effect (32%) in BHK cells (repSDV RSE vs RSE123, Fig. 9B). These findings lend support to the concept that the introduction of the described SINV 3′-UTR motif comprising the three repeated stem-loops strongly enhances, in insect cells, the replicating activity of a virus that lacks these elements. Notably, it has been previously demonstrated that a salmonid alphavirus replicon replicates in cells from fish, mammals and insects in a wide range of temperatures^48^. In addition, it has also been described that a salmonid alphavirus is able to replicate in arthropod cells at low temperatures^49^. To test the influence of temperature on the SDV replicons, we incubated transfected BHK and C6/36 cells at 15 °C for 24 h. Supplementary Fig. S2B shows that luciferase activity at 15 °C was approximately 40–60% lower than that obtained at physiological temperatures for BHK and C6/36 cells (37 °C and 28 °C, respectively). This is presumably due to the fact that the translation machinery is optimal at these temperatures.

To analyze whether the enhanced luciferase activity observed with repSDV RSE was due, at least in part, to a higher translatability of the sgmRNA, we assayed the translation of the different sgmRNAs out of the replication context. Thus, we engineered three new constructs bearing the sgmRNA sequence directly behind the T7 promoter (see scheme of sgSDV in Fig. 9A). The corresponding sgmRNAs were obtained by *in vitro* transcription and transfected into BHK or C6/36 cells. As shown in Fig. 9C (left panel), the presence of SINV repeated sequence elements (sgSDV RSE) did not improve sgSDV translation in BHK cells. In contrast, addition of these elements stimulated luciferase synthesis by 4.5-fold in comparison with sgSDV wt in insect cells (Fig. 9C, right panel). Furthermore, mutation of the inserted sequences (sgSDV RSE123) had a major inhibitory effect (75–80% decrease in luciferase production relative to sgSDV RSE) exclusively in the mosquito cell line (Fig. 9C, right panel). Analysis of RNA levels by qRT-PCR revealed a tendency to a reduced amount of sgSDV RSE and RSE123 as compared to control sgSDV wt containing the genuine SDV 3′-UTR, both in BHK and C6/36 cells at 4 hpt (Fig. 9D). In addition, we did not observe differences when comparing sgSDV RSE with the mutated variant RSE123, suggesting that alterations in Seq 1, Seq 2 and Seq 3 do not affect RNA levels, reinforcing the concept that such sequences are important for translation in mosquito cells. In summary, the addition of the three SINV 3′-UTR repeated sequences results in a more effective replication of repSDV and the corresponding sgmRNA is translated more efficiently in insect cells.

## Discussion

A variety of pseudoknots and/or repeated structures present at the 3′-UTR have been studied in many RNA viruses. In most instances, these structures participate in viral RNA replication since the 3′-UTR is the site at which viral RNA polymerases interact to synthesize the complementary RNA chain^8^. Both g- and sgmRNAs from SINV share the same 3′-UTR and poly(A) tail and thus both regions contain the three repeated stem-loop structures analyzed in this study. For many years, the reason why these repeated regions are conserved not only in several alphaviruses, but also in members of other virus families, has remained an enigma. Consequently, their functioning in viral replication is practically unknown. The fact that they are located at the 3′-UTR most likely oriented investigation towards viral RNA replication rather than mRNA translation. Moreover, our present findings demonstrate that these sequences are required for efficient protein synthesis only in insect cells, and this fact may have added further difficulties to uncover their exact role during the virus life cycle. In this regard, several lines of research on the action of the 3′-UTR of alphaviruses have been documented. First, it has been previously observed that the 3′-UTR repeated elements are not required for the replication of alphavirus RNAs^15^,^23^,^50^,^51^,^52^, suggesting that such regions could have other functions. Interestingly, in Chikungunya virus, the repeated elements present at the 3′-UTR are important for adaptation to mosquitoes rather than to mammalian hosts^40^. Thus, the 3′-UTR constitutes an important trait for the evolution of this virus in invertebrates, but the exact role of the repeated sequences has not been analyzed at the molecular level. Curiously, alphaviruses that replicate only in vertebrates, such as salmon pancreas disease virus and SDV^33^, contain a short 3′-UTR, whereas those restricted to insects, e.g. Eilat virus^53^ or Aura virus^21^,^28^,^54^, carry a longer 3′-UTR. Moreover, deletion of most of the SINV 3′-UTR while retaining the last 19 nt is more deleterious for virus growth in mosquito than in chicken cells^31^. These observations are consistent with the idea that this region may be important for replication in insect cells, but the molecular basis for this behavior had not been previously investigated. Also, some mutations in flaviviruses, such as dengue virus, may hamper its replication in mosquito, but not in mammalian cells^55^,^56^,^57^. Accordingly, specific mutations in the nucleotide sequence but not the structure of one hairpin located at the 3′-UTR of dengue virus genome, negatively affected its replication but not viral translation, selectively in insect cells^56^.

Our findings lend support to the idea that the repeated sequences of SINV 3′-UTR provide translatability to viral mRNAs selectively in mosquito cells. Thus, the motif analyzed in this work would provide an advantage for viral replication in arthropod cells and also account for the modification of the host range of alphaviruses (and perhaps other arboviruses). The present findings may explain, at a molecular level, the acquisition of these repeated regions during alphavirus evolution. It is thought that an alphavirus ancestor initially infected marine organisms and did not have an invertebrate vector^58^. Such an ancestor would not require these repeated elements and would contain a shorter 3′-UTR, as occurs in salmon pancreas disease virus and SDV^36^. Perhaps, the marine alphavirus ancestor adapted at some point in evolution to infect invertebrate hosts. In the present work, we provide experimental evidence for this concept since location of the SINV repeated sequences at the 3′-UTR of an SDV-derived replicon strongly enhances its replication selectively in mosquito cells. This enhancement was also observed in the translation of SDV sgmRNA, as well as in a non-viral mRNA, out of a replication context in insect cells. Along this line, recent evidence suggests that salmonid alphavirus may have the potential to replicate in mosquito cells in a temperature-dependent manner^49^. For this adaptation, the virus might have recombined with another virus present in the vector, such as an insect-borne plant virus as has been proposed^59^, acquiring 3′-UTR elements necessary for translation. It would be interesting in future studies to test whether other alphaviruses, and also other arboviruses, have regions at the 3′-UTR that could provide translatability to their mRNAs selectively in invertebrate hosts. It is also reasonable to think that endogenous insect transcripts might utilize similar elements. For instance, a motif repeated 13 times and located at the 3′-UTR of *oskar* mRNA is required for its translational activation^60^. However, further exploration is necessary to investigate potential motifs at the 3′-UTR of insect mRNAs involved in the regulation of their translation.

In sum, alphaviruses may have evolved two elements that are implicated in the efficient translation of their mRNAs. One such element is the stem-loop structure (DLP) located downstream of the initiation codon of sgmRNA that provides translatability in cells containing PKR after the induction of eIF2α phosphorylation by viral infection^18^,^20^. This structure has been proposed to be responsible for adaptation to certain vertebrate hosts^21^. The second element is the loop sequence present in the repeated hairpins at the 3′-UTR, required for translation of SINV g- and sgmRNA in mosquito cells that do not encode PKR. These observations provide a molecular explanation for the evolutionary bottleneck described for Chikungunya virus and its adaptation to arthropod cells. Additionally, we also examined the hairpin SL1 formed at the start of the sgmRNA of SINV. Our results suggest that this structure is not essential for sgmRNA functioning, but the nucleotide sequence of the loop may be relevant for the efficient synthesis and translation of this mRNA both in mammalian and insect replicating cells, in accord with published observations^22^.

Remarkably, there is no full restoration of translation in the SINV variants described in this work in which the potential base-pairing is reestablished, suggesting that if these sequences are involved in long-range RNA-RNA interactions^37^,^38^, this event does not suffice to restore translation in mosquito cells. This result is in good agreement with findings reported for plant viruses in which kissing-loop interactions have been described^5^. In some instances, alteration of the hairpin present at the 3′-UTR of some plant viruses leads to inhibition of mRNA translation, but protein synthesis is not restored after introducing compensatory mutations in the structure located at the 5′-UTR^7^,^61^,^62^. It may be conceivable that the three stem-loops of SINV 3′-UTR form a superstructure that interacts with the hairpins present at the 5′-ends, which have been already validated by enzymatic and chemical probing analyses^20^,^42^, leading to long-range interactions in g- and/or sgmRNAs. Further RNA structural probing studies aimed at examining the effect of mutations on the global and/or local secondary structure of the UTRs would provide insights into the regulation of translation by these regions. Another plausible hypothesis to account for our findings would be that an insect factor, perhaps a protein or a RNA molecule implicated in translation, binds to a specific sequence and is responsible for the translatability of SINV mRNAs in mosquito cells, as has been suggested^28^,^32^,^56^,^63^,^64^. This hypothesis could also explain other differences found between insect and mammalian cells with respect to SINV sgmRNA translation^17^,^18^,^21^. Future work in this direction will clarify the exact functioning of the different structures and sequences present in alphavirus mRNAs and to what extent analogous mechanisms are operative in other arboviruses.

## Methods

### RNA secondary structure prediction

Minimum free energy secondary structures were predicted using the default parameters of RNAfold WebServer^65^: http://rna.tbi.univie.ac.at/cgi-bin/RNAfold.cgi

### Cell culture

Baby hamster kidney-21 cells (BHK-21, obtained from ATCC) and mouse embryonic fibroblasts (MEFs) derived from *PKR*^−/−^ mice^66^ were grown at 37 °C, 5% CO_2_ in Dulbecco’s modified Eagle’s medium (DMEM) supplemented with 5% fetal calf serum (FCS) or 10% FCS, respectively. *Aedes albopictus* C6/36 cells (obtained from ATCC) were cultured at 28 °C without CO_2_ in M3 medium supplemented with 10% FCS.

### Plasmid construction

The plasmids employed in this work are detailed in Supplementary Table S1. Subgenomic 5′ and 3′-UTRs in SINV replicons were altered by site-directed mutagenesis using the template plasmids and primer sets listed in Supplementary Table S2, and the QuikChange Site-Directed Mutagenesis Kit (Stratagene). To obtain the corresponding sgmRNAs by *in vitro* transcription, plasmids pT7 C+luc 3′/1, pT7 C+luc 3′/2, pT7 C+luc 3′/3, pT7 C+luc 3′/1+2 and pT7 C+luc 3′/1+2+3 were generated by inserting the SacI/AatII-digested PCR product obtained with pT7 rep C+luc^67^ as DNA template and oligonucleotides 5′SacI-T7prom and 3′Aat, into the equivalent sites of the plasmids described in Supplementary Table S2. The plasmid pT7 C+luc sg5′/12–18 was constructed using the plasmid pT7 rep C+luc sg5′/12–18 as template in a PCR reaction with oligonucleotides 5′SacI-T7prom and 3′Aat. The PCR product was digested with SacI and AatII restriction endonucleases and cloned into the equivalent sites of pT7 rep C+luc. The plasmid pT7 C+luc sg5′/12–18 + 3′/1+2+3 was made by moving the SacI/AatII-digested product from pT7 C+luc sg5′/12–18 into pT7 C+luc 3′/1+2+3. To obtain non-replicating SINV genomic RNA with mutated 5′ and 3′-UTRs by *in vitro* transcription, the following plasmids were engineered: pToto1101/Luc ∆nsP4, pToto1101/Luc ∆nsP4 3′/1+2 and pToto1101/Luc ∆nsP4 3′/1+2+3 were constructed by digesting the plasmids pT7 rep C+luc, pT7 rep C+luc 3′/1+2 and pT7 rep C+luc 3′/1+2+3, respectively, with EcoNI (blunt) and XhoI restriction endonucleases. Digested products, containing the 3′-UTR, were then inserted into pToto1101/Luc^68^ using XhoI and HpaI/blunt restriction sites. The plasmids pToto1101/Luc ∆nsP4 g5′/12–18 and pToto1101/Luc ∆nsP4 g5′/12–18 + 3′/1+2+3 were generated by the insertion of a double PCR product digested with SacI and AfeI into the corresponding sites of pToto1101/Luc ∆nsP4 and pToto1101/Luc ∆nsP4 3′/1+2+3, respectively. We performed double amplifications as follows: first, we performed two PCRs using primers 5′ gen7 and 3′ AfeI-gen7 or primers 5′ SacI-gen7 and 3′ gen7 with pToto1101/Luc as DNA template. Then we used a mixture of these products and the primers 5′ SacI-gen7 and 3′ AfeI-gen7 for the second PCR. The plasmid pT7 Lluc-luc was constructed using the plasmid pT7 rep Lluc-luc^69^ as DNA template in a PCR reaction with oligonucleotides 5′SacI-T7prom and 3′Luc SphI. PCR products were then digested with SacI and SphI restriction endonucleases and transferred to the same sites of pT7 rep Lluc-luc. The plasmid pT7 Lluc-luc 3′/1+2+3 was obtained by inserting a DNA fragment corresponding to the mutated 3′-UTR from pT7 rep C+luc 3′/1+2+3 into pT7 Lluc-luc, between the EcoNI and XhoI sites. The plasmid pnsP-LUC^35^ was a generous gift from Dr. M. Brémont (INRA, Jouy en Josas, France). The plasmids pnsP-LUC 3′/RSE and pnsP-LUC 3′/RSE 1+2+3 were prepared as follows. First, we carried out a PCR reaction with pT7 rep C+luc or pT7 rep C+luc 3′/1+2+3, respectively, as DNA template and primers 5′EcoRV-RSE and 3′RSE-SDV. Next, pnsP-LUC was employed as DNA template in a second PCR with primers 5′SDV and 3′SDV. Both products were then mixed to be used as templates in a new PCR with oligonucleotides 5′EcoRV-RSE and 3′SDV. The resulting double PCR products were inserted between the EcoRV and XbaI sites of the pnsP-LUC vector. Finally, to synthesize the corresponding sgmRNAs by *in vitro* transcription, we engineered the plasmids pT7 SDV-LUC, pT7 SDV-LUC 3′/RSE and pT7 SDV-LUC 3′/RSE 1+2+3 by cloning the EcoRI/XbaI-digested PCR product obtained with pnsP-LUC as DNA template and primers 5′EcoRI-T7-SDV and 3′SDV, into the equivalent sites of pnsP-LUC, pnsP-LUC 3′/RSE and pnsP-LUC 3′/RSE 1+2+3, respectively. All plasmid constructions were verified by DNA sequencing.

### *In vitro* transcription and transfection

Plasmids were linearized with XhoI (or NotI in the case of all plasmids derived from pnsP-LUC) and used as templates for *in vitro* RNA transcription with T7 or SP6 RNA polymerase (New England Biolabs). Reactions containing 12.5 ng/μl of template DNA, 1000 U/ml of RNA polymerase, 1× RNAPol Reaction Buffer, 1 mM m^7^G(5′)ppp(5′)G RNA Cap Structure Analog (New England Biolabs), 0.5 mM ATP, 0.5 mM CTP, 0.5 mM UTP, 0.25 mM GTP, and 0.32 U/μl RNaseOUT Recombinant Ribonuclease Inhibitor (Invitrogen) were incubated for 4 h at 37 °C. The template DNA was then digested with 0.1 U/μl RNase-free Recombinant DNase I (Takara) for 10 minutes at 37 °C. RNAs synthesized *in vitro* were purified using the RNeasy Mini Kit (Qiagen) and quantified with the Nanodrop ND-1000 spectrophotometer. For transfection, cells were grown to 70–80% confluence in 24-well plates with antibiotic- and antimycotic-free DMEM or M3 supplemented with 10% FCS. A mixture containing 2 μl of Lipofectamine 2000 reagent (Invitrogen) and 50 μl of Opti-MEM I Reduced Serum Medium (Gibco) was incubated for 5 minutes at room temperature. Simultaneously, 2 μg of *in vitro* synthesized RNA were added to 50 μl of Opti-MEM I for each L-24 well and incubated at room temperature for 5 minutes. The final transfection mixture was subsequently prepared with 50 μl of the Lipofectamine suspension and 50 μl of the RNA mixture by incubation for 30 minutes at room temperature. Cell medium was then replaced by 150 μl of Opti-MEM I plus 100 μl of the transfection mixture per well and cells were incubated for 2 h at 28 °C (C6/36 cells) or 37 °C (BHK cells). Next, lipofectamine-containing medium was removed and the cultures continued in fresh medium containing 10% FCS.

### Determination of luciferase activity

For firefly luciferase assays, cells were recovered in lysis buffer (0.5% Triton X-100, 25 mM glycylglycine pH 7.8 and 1 mM dithiothreitol) and luciferase activity was measured using the Luciferase Assay System (Promega). *Renilla* luciferase activity was detected using the *Renilla* Luciferase Assay System (Promega).

### Data analysis

Statistical analysis was performed with IBM SPSS Statistics 21. The normality of the data was analyzed by Shapiro-Wilk test. P-values relative to wt mRNAs were calculated using two-tailed Student’s t-test (data with normal distribution) or Mann-Whitney U-test (data with non-normal distribution). Data were considered significant if P<0.05 (*P<0.05; **P<0.01; ***P<0.001).

### Metabolic labeling

Radioactive labeling of proteins was performed in methionine/cysteine-free DMEM supplemented with [^35^S]Met/Cys (EasyTag^TM^ Express ^35^S Protein Labeling mix, Perkin Elmer). Samples were then collected in sample buffer (0.37 M Tris-HCl pH 6.8, 0.1 M DTT, 2% SDS, 17% glycerol and 0.024% bromophenol blue), boiled for 5 minutes and analyzed by autoradiography of SDS-polyacrylamide gels (15%). To analyze the synthesis of viral RNA, cells were treated with actinomycin D (5 μg/ml) from 2 hpt and with [^3^H]uridine (40 μCi/ml, final concentration) from 3 hpt. Total RNA was extracted at 7 hpt using the RNeasy Mini Kit (Qiagen) and subjected to electrophoresis in 0.7% agarose-phosphate gels following glyoxal and dimethyl sulfoxide denaturation^70^. The gel was then dried and exposed to X-ray film at −70 °C.

### Immunoblotting

Cells were harvested in sample buffer, boiled, processed by SDS-PAGE and transferred to a nitrocellulose membrane for Western blot analysis using rat anti-nsP1 polyclonal antibodies^71^, rabbit anti-nsP2 polyclonal antibodies (a kind gift from Dr. R. W. Hardy, Indiana University, USA) and total eIF2α (Santa Cruz Biotechnology).

### Quantitative reverse-transcription RT-PCR

Total RNA was extracted from transfected BHK or C6/36 cells at 4 hpt using the RNeasy Mini Kit (Qiagen). Then, 500 ng RNA was reverse transcribed using the iScript cDNA Synthesis kit (BioRad). The resulting cDNAs were subjected to qRT-PCR using Power SYBR Green (Applied Biosystems) with C, firefly luciferase and *Renilla* luciferase specific primers (SubG4, luc2 and RLUC1, respectively, in Supplementary Table S2). PCR amplification was performed by incubation at 95 °C for 10 minutes followed by 40 cycles at 95 °C for 15 s and 60 °C for 60 s, and a final step for 15 s at 95 °C + 15 s at 60 °C + 15 s at 95 °C, using the CFX384 Touch Real-Time PCR Detection System (BioRad). Values were normalized in BHK cells with the β-Actin and lysophosphatidic acid receptor-4 internal controls; in C6/36 cells, normalization was performed with the La autoantigen homolog and ribosomal protein S6 internal controls. For these reactions, we used the specific primers detailed in Supplementary Table S2 and the same conditions as before. The specificity of the amplification reactions was confirmed by analyzing the corresponding melting curves. Data analysis was carried out using GenEx software (version 6).

### *In vitro* translation

Nuclease-treated Rabbit Reticulocyte Lysate system (RRL, Promega) was used for *in vitro* translation. Reactions containing 100 ng of *in vitro* transcribed mRNAs were incubated for 90 minutes at 30 °C. An *in vitro* translation assay in *Drosophila* embryo extracts was carried out as previously described^47^. Reaction mixtures containing 200 ng of *in vitro* synthesized RNA, 40% embryo extract (a kind gift from Dr. F. Gebauer, Centre for Genomic Regulation, Barcelona, Spain), 0.1 mM spermidine, 60 μM amino acids, 16.8 mM creatine phosphate, 80 ng/μl creatine kinase, 24 mM HEPES pH 7.4, 1.4 mM magnesium acetate, 100 mM potassium acetate and 100 ng/μl calf liver tRNA were incubated in a final volume of 10 μl at 25 °C for 90 minutes. Protein synthesis was determined by measuring luciferase activity.

## Additional Information

**How to cite this article**: Garcia-Moreno, M. *et al.* A Viral mRNA Motif at the 3'-Untranslated Region that Confers Translatabilityin a Cell-Specific Manner. Implications for Virus Evolution. *Sci. Rep.* **6**, 19217; doi: 10.1038/srep19217 (2016).

## Supplementary Material

Supplementary Information
