# Supplementary material for: A Viral mRNA Motif at the 3′-Untranslated Region that Confers Translatability in a Cell-Specific Manner. Implications for Virus Evolution
Source: Sci Rep. 2016 Jan 12;6:19217. doi: 10.1038/srep19217 (PMC4709744; doi:10.1038/srep19217)
Supplement: Supplementary Information [file srep19217-s1.pdf]

SUPPLEMENTARY INFORMATION

A Viral mRNA Motif at the 3′-Untranslated Region that Confers Translatability in a Cell-Specific Manner. Implications for Virus Evolution.

Manuel Garcia-Moreno, Miguel Angel Sanz and Luis Carrasco

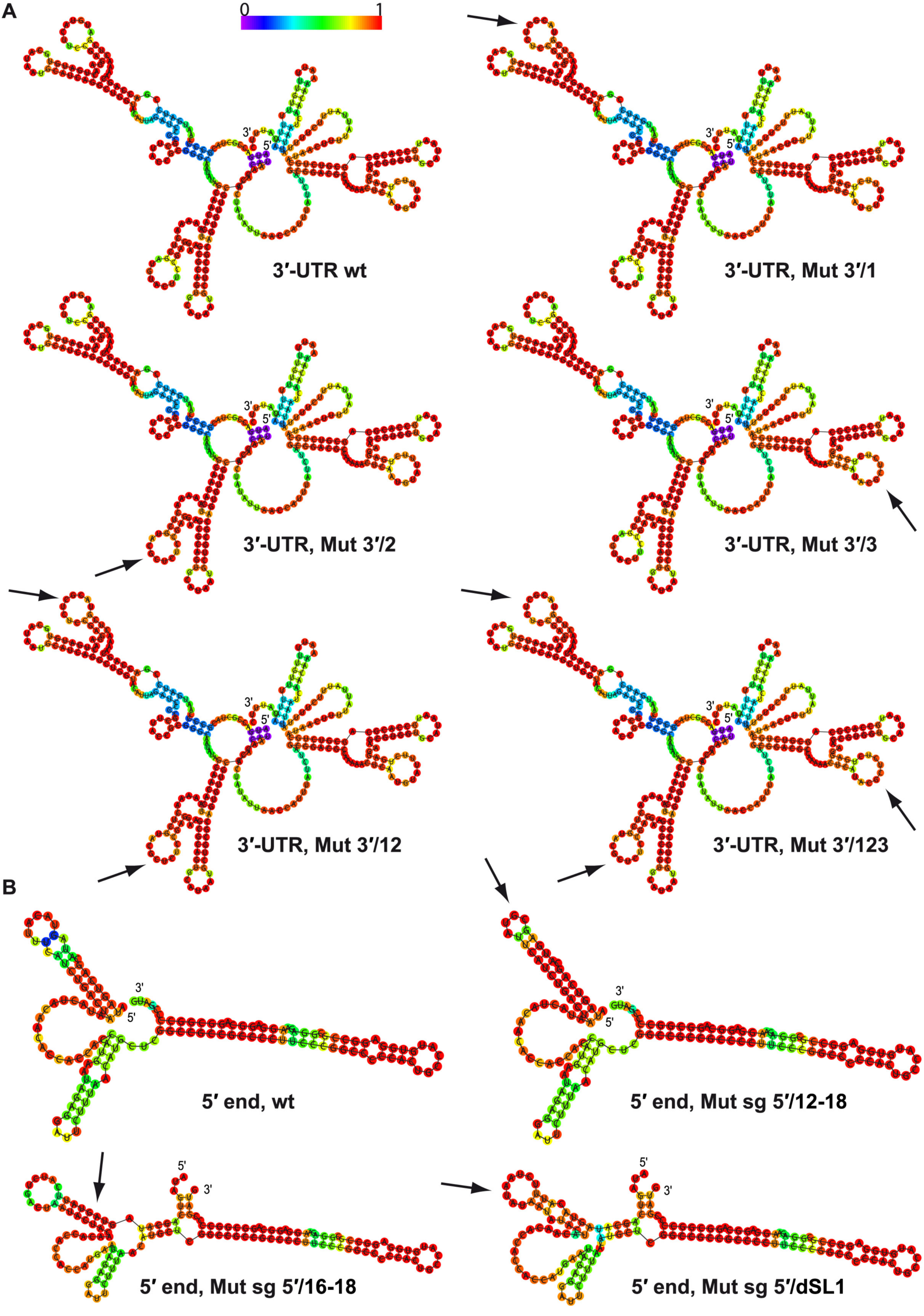

A
0
1
3′-UTR wt
3′-UTR, Mut 3′/1
3′-UTR, Mut 3′/2
3′-UTR, Mut 3′/3
3′-UTR, Mut 3′/12
3′-UTR, Mut 3′/123
B
5′ end, wt
5′ end, Mut sg 5′/12-18
5′ end, Mut sg 5′/16-18
5′ end, Mut sg 5′/dSL1

Supplementary Figure S1. Base-pairing probabilities of the 3′ and 5′ ends of different SINV RNAs. The minimum free energy secondary structure of the SINV 3′-UTRs (*A*) and sgmRNA 5′ ends (*B*) generated in this work were predicted using the RNAfold WebServer. Colors denote base-pairing probabilities or, in the case of unpaired bases, the probability of being unpaired. Arrows indicate the location of the different mutations (see more details in Fig. 1).

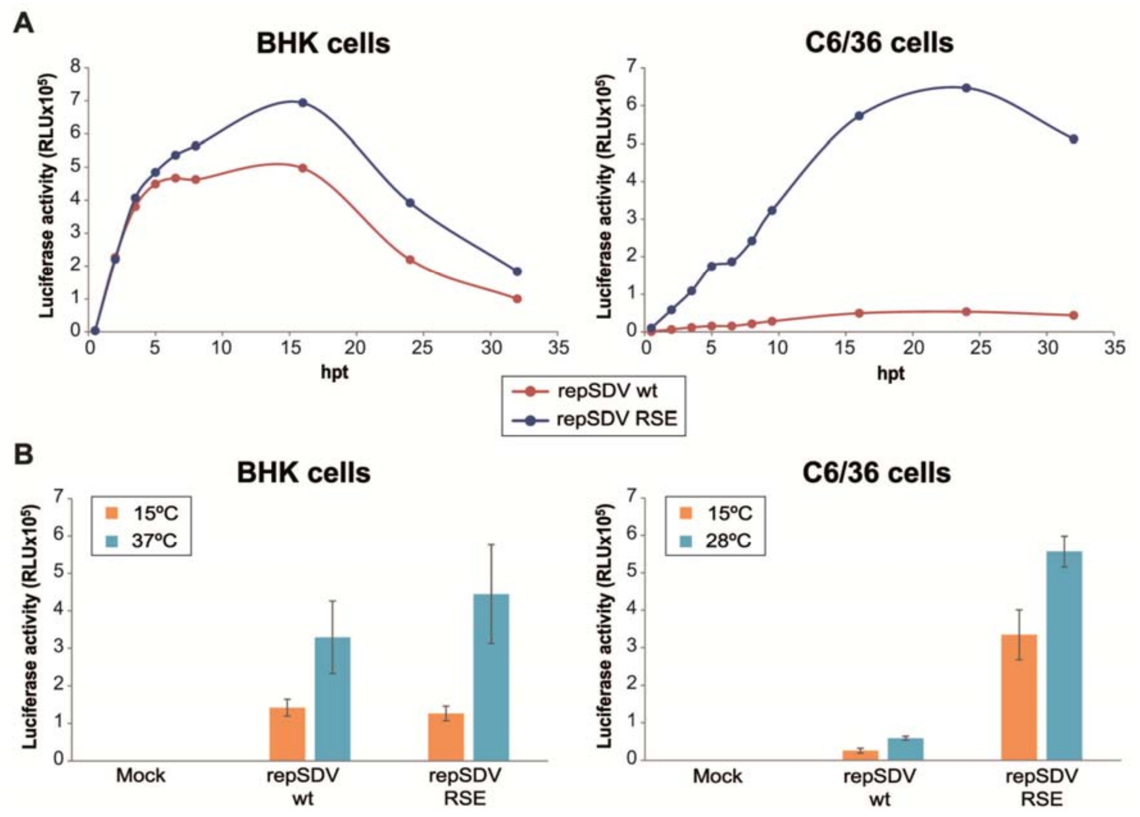

Supplementary Figure S2. Production of luciferase from SDV replicons at different times and temperatures. (*A*) BHK (*left panel*) and C6/36 (*right panel*) cells were transfected with repSDV wt or repSDV RSE using Lipofectamine 2000. At different times post-transfection, cells were harvested and lysed to measure *Renilla* luciferase activity. (*B*) The SDV replicons were transfected into BHK (*left panel*) or C6/36 (*right panel*) cells using Lipofectamine 2000 and incubated at the indicated temperatures for 24 h. Next, *Renilla* luciferase activity was determined. Values are represented as means ± SD of two representative experiments.

Supplementary Table S1. Nomenclature of plasmids, replicons, sgmRNAs and gmRNAs, with their respective abbreviated names.

Plasmid	replicon/gmRNA	sgmRNA	Abbreviated name	Product obtained by *in vitro* transcription
pT7 rep C+luc	rep C+luc	C+luc	rep wt	SINV replicon bearing the C gene followed by the luc gene.
pT7 rep C+luc sg5′/12-18	rep C+luc sg5′/12-18	C+luc sg5′/12-18	rep 5′/12-18	SINV replicon bearing the C gene followed by the luc gene. The nucleotides 12 to 18 of the sg 5′-UTR are mutated.
pT7 rep C+luc sg5′/16-18	rep C+luc sg5′/16-18	C+luc sg5′/16-18	rep 5′/16-18	SINV replicon bearing the C gene followed by the luc gene. The nucleotides 16 to 18 of the sg 5′-UTR are mutated.
pT7 rep C+luc sg5′/dSL1	rep C+luc sg5′/dSL1	C+luc sg5′/dSL1	rep 5′/dSL1	SINV replicon bearing the C gene followed by the luc gene. The SL1 structure of the sg 5′-UTR is disrupted.
pT7 rep C+luc 3′/1	rep C+luc 3′/1	C+luc 3′/1	rep 3′/1	SINV replicon bearing the C gene followed by the luc gene. Seq 1 of the 3′-UTR is mutated.
pT7 rep C+luc 3′/2	rep C+luc 3′/2	C+luc 3′/2	rep 3′/2	SINV replicon bearing the C gene followed by the luc gene. Seq 2 of the 3′-UTR is mutated.
pT7 rep C+luc 3′/3	rep C+luc 3′/3	C+luc 3′/3	rep 3′/3	SINV replicon bearing the C gene followed by the luc gene. Seq 3 of the 3′-UTR is mutated.
pT7 rep C+luc 3′/1+2	rep C+luc 3′/1+2	C+luc 3′/1+2	rep 3′/12	SINV replicon bearing the C gene followed by the luc gene. Seq 1 and Seq 2 of the 3′-UTR are mutated.
pT7 rep C+luc 3′/1+2+3	rep C+luc 3′/1+2+3	C+luc 3′/1+2+3	rep 3′/123	SINV replicon bearing the C gene followed by the luc gene. Seq 1, Seq 2 and Seq 3 of the 3′-UTR are mutated.
pToto1101/Luc ΔnsP4	SV-luc ΔnsP4 wt		G wt	Recombinant SINV containing the luc gene placed inside the nsP3 sequence. nsP4 is cleaved at the HpaI restriction site. This mRNA is unable to replicate.
pToto1101/Luc ΔnsP4 g5′/12-18	SV-luc ΔnsP4 g5′/12-18		G 5′/12-18	Recombinant SINV containing the luc gene placed inside the nsP3 sequence. nsP4 is cleaved at the HpaI restriction site. This mRNA is unable to replicate. The nucleotides 12 to 18 of the g 5′-UTR are mutated.

Plasmid	replicon/gmRNA	sgmRNA	Abbreviated name	Product obtained by *in vitro* transcription
pToto1101/Luc ΔnsP4 3′/1+2	SV-luc ΔnsP4 3′/1+2		G 3′/12	Recombinant SINV containing the luc gene placed inside the nsP3 sequence. nsP4 is cleaved at the HpaI restriction site. This mRNA is unable to replicate. Seq 1 and Seq 2 of the 3′-UTR are mutated.
pToto1101/Luc ΔnsP4 3′/1+2+3	SV-luc ΔnsP4 3′/1+2+3		G 3′/123	Recombinant SINV containing the luc gene placed inside the nsP3 sequence. nsP4 is cleaved at the HpaI restriction site. This mRNA is unable to replicate. Seq 1, Seq 2 and Seq 3 of the 3′-UTR are mutated.
pToto1101/Luc ΔnsP4 g5′/12-18 + 3′/1+2+3	SV-luc ΔnsP4 g5′/12-18 + 3′/1+2+3		G 5′+3′	Recombinant SINV containing the luc gene placed inside the nsP3 sequence. nsP4 is cleaved at the HpaI restriction site. This mRNA is unable to replicate. The nucleotides 12 to 18 of the g 5′-UTR and Seq 1, Seq 2 and Seq 3 of the 3′-UTR are mutated.
pT7 C+luc		C+luc wt	SG wt	SINV sgmRNA bearing the C gene followed by the luc gene. This mRNA is unable to replicate.
pT7 C+luc sg5′/12-18		C+luc sg5′/12-18	SG 5′/12-18	SINV sgmRNA bearing the C gene followed by the luc gene. This mRNA is unable to replicate. The nucleotides 12 to 18 of the sg 5′-UTR are mutated.
pT7 C+luc 3′/1		C+luc 3′/1	SG 3′/1	SINV sgmRNA bearing the C gene followed by the luc gene. This mRNA is unable to replicate. Seq 1 of the 3′-UTR is mutated.
pT7 C+luc 3′/2		C+luc 3′/2	SG 3′/2	SINV sgmRNA bearing the C gene followed by the luc gene. This mRNA is unable to replicate. Seq 2 of the 3′-UTR is mutated.
pT7 C+luc 3′/3		C+luc 3′/3	SG 3′/3	SINV sgmRNA bearing the C gene followed by the luc gene. This mRNA is unable to replicate. Seq 3 of the 3′-UTR is mutated.
pT7 C+luc 3′/1+2		C+luc 3′/1+2	SG 3′/12	SINV sgmRNA bearing the C gene followed by the luc gene. This mRNA is unable to replicate. Seq 1 and Seq 2 of the 3′-UTR are mutated.
pT7 C+luc 3′/1+2+3		C+luc 3′/1+2+3	SG 3′/123	SINV sgmRNA bearing the C gene followed by the luc gene. This mRNA is unable to replicate. Seq 1, Seq 2 and Seq 3 of the 3′-UTR are mutated.
pT7 C+luc sg5′/12-18 + 3′/1+2+3		C+luc sg5′/12-18 + 3′/1+2+3	SG 5′+3′	SINV sgmRNA bearing the C gene followed by the luc gene. This mRNA is unable to replicate. The nucleotides 12 to 18 of the sg 5′-UTR and Seq 1, Seq 2 and Seq 3 of the 3′-UTR are mutated.

Plasmid	replicon/gmRNA	sgmRNA	Abbreviated name	Product obtained by *in vitro* transcription
pT7 Lluc-luc		Lluc-luc wt	luc wt	mRNA encoding the luc gene under the control of the luc leader sequence. Contains SINV 3′-UTR.
pT7 Lluc-luc 3′/1+2+3		Lluc-luc 3′/1+2+3	luc 3′/123	mRNA encoding the luc gene under the control of the luc leader sequence. Contains SINV 3′-UTR with mutated Seq 1, Seq 2 and Seq 3.
pnsP-LUC	rep SDV-LUC wt	SDV-LUC wt	repSDV wt	SDV replicon bearing the LUC gene in place of the structural coding region.
pnsP-LUC 3′/RSE	rep SDV-LUC 3′/RSE	SDV-LUC 3′/RSE	repSDV RSE	SDV replicon bearing the LUC gene in place of the structural coding region. Contains the RSE of SINV at the beginning of the 3′-UTR.
pnsP-LUC 3′/RSE 1+2+3	rep SDV-LUC 3′/RSE 1+2+3	SDV-LUC 3′/RSE 1+2+3	repSDV RSE123	SDV replicon bearing the LUC gene in place of the structural coding region. Contains the RSE of SINV with mutated Seq 1, Seq 2 and Seq 3 at the beginning of the 3′-UTR.
pT7 SDV-LUC		SDV-LUC wt	sgSDV wt	SDV sgmRNA bearing the LUC gene in place of the structural coding region. This mRNA is unable to replicate.
pT7 SDV-LUC 3′/RSE		SDV-LUC 3′/RSE	sgSDV RSE	SDV sgmRNA bearing the LUC gene in place of the structural coding region. This mRNA is unable to replicate. Contains the RSE of SINV at the beginning of the 3′-UTR.
pT7 SDV-LUC 3′/RSE 1+2+3		SDV-LUC 3′/RSE 1+2+3	sgSDV RSE123	SDV sgmRNA bearing the LUC gene in place of the structural coding region. This mRNA is unable to replicate. Contains the RSE of SINV with mutated Seq 1, Seq 2 and Seq 3 at the beginning of the 3′-UTR.

Abbreviations: *gmRNA,* genomic mRNA; *sgmRNA,* subgenomic mRNA; *SINV,* Sindbis virus; *C,* capsid protein; *luc,* firefly luciferase; *sg*, subgenomic; *UTR,* untranslated region; *SL*, stem-loop; Seq, sequence; *nsP,* non-structural protein; *g*, genomic; *SDV*, Sleeping disease virus; *LUC*, *Renilla* luciferase; *RSE*, repeated sequence elements.

Supplementary Table S2. List of plasmids, template plasmids and primers.

Plasmid	Template plasmid	Primers	5′-3′ Sequence
pT7 rep C+luc sg5′/12-18	pT7 rep C+luc	5′ 7nts	ATAGTCAGCATGAGCGTATTCATCTGACTAATAC
		3′ 7nts	GTATTAGTCAGATGAATACGCTCATGCTGACTAT
pT7 rep C+luc sg5′/16-18	pT7 rep C+luc	5′ 3nts	ATAGTCAGCATAGTAGTATTCATCTGACTAATAC
		3′ 3nts	GTATTAGTCAGATGAATACTACTATGCTGACTAT
pT7 rep C+luc sg5′/dSL1	pT7 rep C+luc	5′ dSL1	CAGCATAGTACATTTCTAATATAATATACTACAACACC
		3′ dSL1	GGTGTTGTAGTATATTATATTAGAAATGTACTATGCTG
pT7 rep C+luc 3′/1	pT7 rep C+luc	5′ Mut1	GATCCGACCAGCAAAACTCGTACGCTCTCCGAGGAAC TGATGTGCATAATGCATCAGGCTGGTACATTAG
		3′ Mut1	GATCTAATGTACCAGCCTGATGCATTATGCACATCAGT TCCTCGGAGAGCGTACGAGTTTTGCTGGTCGGATCAT TGGGGC
pT7 rep C+luc 3′/2	pT7 rep C+luc	5′ Mut2	GCAATATAGCAACACTAAAAACTCGTACGCTCTCCGAG GAAGCGCAGTGCATAATGCTGCGCAGTGTTGCCACAT AACCAC
		3′ Mut2	CAACACTGCGCAGCATTATGCACTGCGCTTCCTCGGA GAGCGTACGAGTTTTTAGTGTTGCTATATTGCCCG
pT7 rep C+luc 3′/3	pT7 rep C+luc	5′ Mut3	CATTTATCTAGCGGACGCCAAAAACTCATACGCTCTCT GAGGAAGCGTGGTGCATAATGCCACGCAGCGTCTGCA TAAC
		3′ Mut3	GTTATGCAGACGCTGCGTGGCATTATGCACCACGCTT CCTCAGAGAGCGTATGAGTTTTTGGCGTCCGCTAGAT AAATG
pT7 rep C+luc 3′/1+2	pT7 rep C+luc 3′/1	5′ Mut2	
		3′ Mut2	
pT7 rep C+luc 3′/1+2+3	pT7 rep C+luc 3′/1+2	5′ Mut3	
		3′ Mut3	
		5′SacI-T7prom	GCGCGCGAGCTCTAATACGACTCACTATAGATAGTCA GCATAGT
		3′Aat	CGTTCTTGACGTCGAAC
		5′gen7	GACGGCGTGAGCGTAACTATTGAATC
		3′AfeI-gen7	CATGGGAAAAGCGCTAAAAGAGGCTGGG
		5′SacI-gen7	CGGAGCCTATGGAAAAACGCCAGC
		3′gen7	CAATAGTTACGCTCACGCCGTCAATC
		3′Luc SphI	CCCGGGGCATGCGAGAATCTGACGCAG
		5′EcoRV-RSE	TGAACAATAAGATATCCGCTACGCCCCAATGATCCGAC C

Primers	5′-3′ Sequence
3′RSE-SDV	GATGGGCTCCTAGTGAGCGATTTGTATACCGGAATGTTATGCAGACGCTGCGTGGCATTATG
5′SDV	ATTCCGGTATACAAATCGCTCACTAGGAGCCCATC
3′SDV	GGCTGATCAGCGAGCTCTAGC
5′EcoRI-T7-SDV	GAGGGCGAATTCTAATACGACTCACTATAGTAACCTTAATATTTTCTGC
Left SubG4	ATCCCTCGCGGAGTAGGA
Right SubG4	ACCGGAGTTATCCATGATCG
Left luc2	AGGTCTTCCCGACGATGA
Right luc2	GTCTTTCCGTGCTCCAAAAC
Left RLUC1	GGAGAATAACTTCTTCGTGGAAAC
Right RLUC1	GCTGCAAATTCTTCTGGTTCTAA
Left La1	CGTGTACGTGAAAGGATTTGC
Right La1	AACTCGAGCAGTTCGCTCAT
Left RPS2	AACGACAAGCAGGGTTTCC
Right RPS2	CGTGGGCGATAGCAAGAG
Left Act2	CTCTCCCTCATGCCATCCT
Right Act2	GTAGCCACGCTCGGTCAG
Left LPAR2	CCTTCCGATCTCGTACCATT
Right LPAR2	GGATCCAGACTCCAGCACAT
